# Long non-coding RNA CCAT1 promotes colorectal cancer progression by regulating miR-181a-5p expression

**DOI:** 10.18632/aging.103139

**Published:** 2020-05-07

**Authors:** Anquan Shang, Weiwei Wang, Chenzheng Gu, Wei Chen, Wenying Lu, Zujun Sun, Dong Li

**Affiliations:** 1 Department of Laboratory Medicine, Tongji Hospital of Tongji University School of Medicine, Shanghai, 200065, China; 2 Department of Pathology, The Sixth People’s Hospital of Yancheng, Yancheng 224001, Jiangsu, China

**Keywords:** colorectal cancer, lncRNA CCAT1, miR-181a-5p, proliferation, apoptosis

## Abstract

The vital roles of long noncoding RNAs (lncRNAs) have been implicated in growing number of studies in tumor development. LncRNA CCAT1 has been recognized as associated with tumor development, yet its relation with colorectal cancer (CRC) remains elusive. Our study aimed at elucidating the function and mechanisms of long non-coding RNA CCAT1 in CRC. From a lncRNA profile dataset of 38 pairs of matched tumor-control colon tissues from colorectal patients housed in The Cancer Genome Atlas (TCGA), we detected 10 upregulated and 10 down-regulated lncRNAs in CRC. Fifty cases of CRC patients were enrolled to analyze the correlation between the expression of CCAT1 and clinical pathology. The inverse correlation of expression and target relationship between CCAT1 and miR-181a-5p were verified using qRT-PCR and dual-luciferase reporter gene assay. Cell viability, colony formation ability, aggression and apoptosis were determined by MTT assay, colony formation assay, Transwell and wound healing assays and flow cytometry analysis. Furthermore, Xenograft model was used to show that knockdown of CCAT1 inhibits tumor growth in vivo. The expression of lncRNA CCAT1 was significantly upregulated in CRC tissues. The CCAT1 expression was positively associated with cancer stage (American Joint Committee on Cancer stage, *P*<0.05). CCAT1 promoted cell proliferation, growth and mobility by targeting miR-181a-5p and the silence of CCAT1 increased the cell apoptosis. Same effect was observed in an in vivo xenograft model, which the tumor size and pro-tumor proteins were significantly diminished by knocking down of CCAT1.

## INTRODUCTION

Colorectal cancer (CRC) is the third most fatal malignant neoplasm worldwide [1]. Research has been done and genetic or epigenetic abnormalities have been related to CRC tumorigenesis and progression [2]. Tumor progression and metastasis are the main cause of death in CRC patients, especially in those at later stages. Despite continuous improvement in diagnostic and medical techniques with various anti-cancer drugs, the overall survival of CRC patients remains comparatively low. Therefore, it is aspired to explore the de facto pathophysiological mechanisms underlying the development of CRC, which promises early diagnosis and therapy of CRC.

Long noncoding RNAs (lncRNAs), >200 nucleotides in length, are newly discovered non-coding RNA molecules that are rarely translated into proteins [3]. Recently, many lncRNAs are shown to regulate the progression of important tumors, including the ability of proliferation, apoptosis, metastasis, senescence, metabolism and drug-resistance [4]. To date, although a number of lncRNAs have been functionally denominated, a majority of them remain unrecognized and warrant further research.

Colon-cancer-associated transcript-1 (CCAT1), a 2,628-bp lncRNA located on chromosome 8q24.21, is initially expressed in CRC and contributes to the tumor progression [5]. Several lines of evidence have confirmed that CCAT1 was singularly expressed and promoted carcinogenesis in various types of tumors, such as gastric cancer [6], hepatocellular carcinoma [7], renal cell carcinoma [8] and lung cancer [9]. Recently, CCAT1 was revealed to act as an oncogene and promote CRC progression [10].

Micro RNAs (miRNAs) are highly conserved noncoding RNAs that have been implicated in the modulating the proliferation, invasion, migration, death, apoptosis or drug resistance by downregulating proteins that promote or suppress tumors [11, 12]. Members of the miR-181 family are found dysregulated in CRCs. The miR-181 family is highly conserved and consists of four members, including miR-181a-5p, miR-181b, miR-181c, and miR-181d in both humans and mice [13]. Ectopic expression of miR-181a-5p has been found in a plethora of human neoplasms, which is significantly associated with the clinical outcomes of cancer patients. MiR-181a-5p is usually under-expressed in cancers, indicating its potential role as a cancer suppressor such as non-small cell lung cancer cell proliferation [14]. Du et al. discovered up-regulation of microRNA-181a-5p in melanoma cells could induce apoptosis [15]. Han et al. observed that miR-181a-5p was downregulated in colorectal cancer [16]. Beyond these, other cancers such as gastric carcinoma [17] and cutaneous squamous cell carcinoma [18] have also been studied.

Herein we found significantly overexpressed lncRNA CCAT1 in human CRC tissue samples. We identified miR-181a-5p as a target of CCAT1, and the expression of miR-181a-5p was inhibited by CCAT1. Knockdown of CCAT1 and overexpression of miR-181a-5p in CRC cell lines both inhibited cell proliferation and increased apoptosis. Taken together, our study demonstrated that lncRNA CCAT1 could regulate the progression of CRC via down-regulating the expression levels of miR-181a-5p.

## RESULTS

### CCAT1 was upregulated in CRC tissues

**Figure 1:**
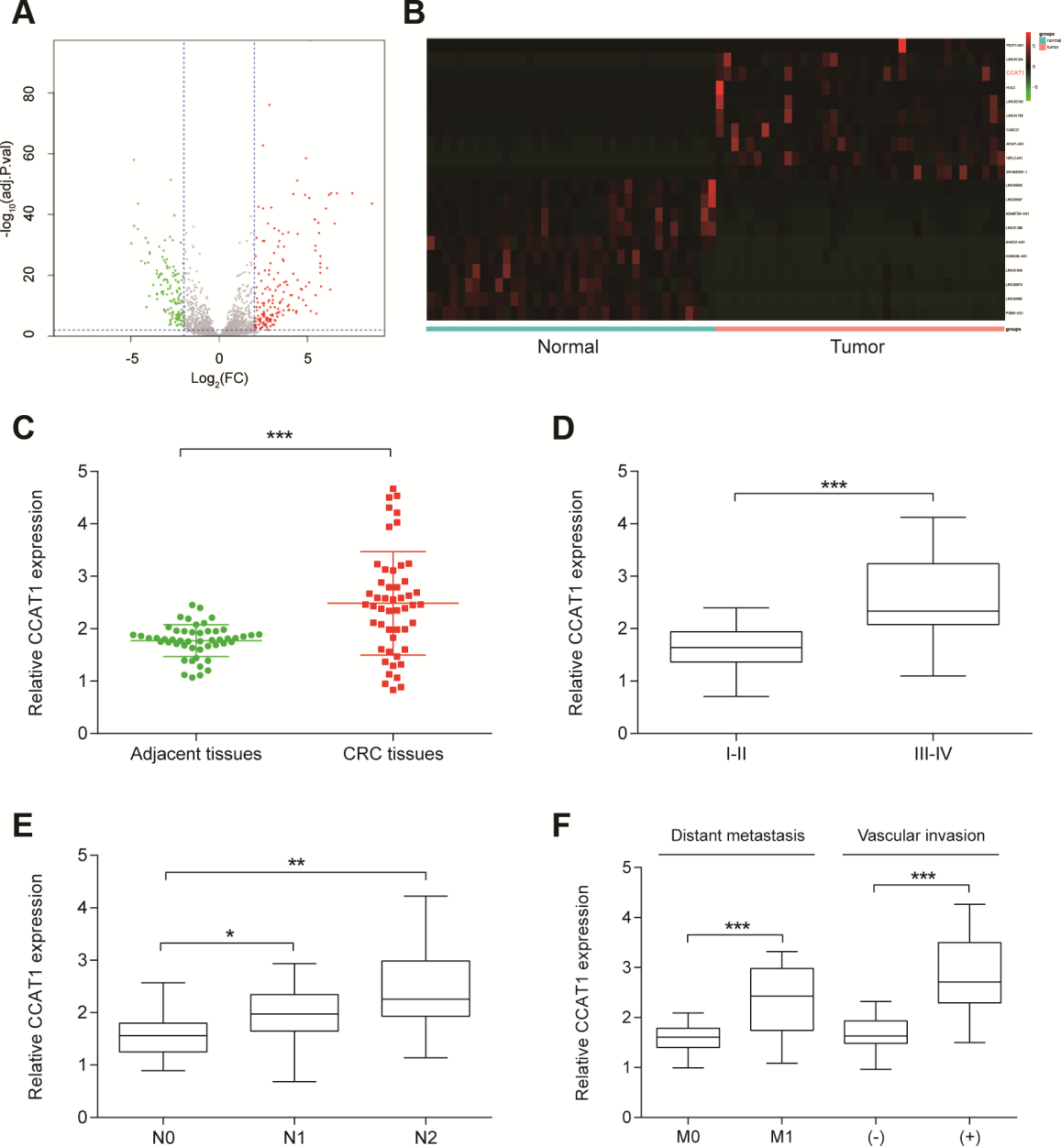
**CCAT1 was overexpressed in CRC tissues.** (**A**) The volcano plot showed the relationship between fold change and significance of lncRNA expression. (**B**) The heat map of 10 high-expressed lncRNAs and 10 low-expressed ones in CRC tissues. (**C**) The expression level of CCCAT1 in 50 CRC tissues was significantly higher, compared with 50 matched adjacent tissues. (**D**–**F**) CCAT1 expression was significantly higher in 14 patients with advanced stage (III-IV), 29 patients with lymph node metastasis, 24 patients with vascular invasion and 12 patients with distant metastasis. All assays were performed three times. ^*^P<0.05, ^**^P<0.01, ^***^P <0.001.

A total of 178 upregulated lncRNAs and 141 downregulated lncRNAs were identified in 38 pairs CRC tissues. Top 10 upregulated including CCAT1 and downregulated lncRNAs were listed (Figure 1A, 1B, Table 1). Compared with matched adjacent normal tissues, CCAT1 expression in CRC tissues was significantly increased (*P*<0.001, Figure 1C). In addition, to investigate the clinical significance of CCAT1, the correlations between its expression level and clinicopathological characteristics were analyzed (Table 2). The analysis results showed that CCAT1 expression level was positively associated with advanced stage, lymph node metastasis, distant metastasis or vascular invasion (*P*<0.05, Figure 1D–1F).

**Table 1:** Detailed data of top 10 up- and down-regulated lncRNA.

**Gene symbol**	**log_2_FC**	**P.value**	**FDR**
PGM5-AS1	-5.1072	1.45E-32	6.04E-31
LINC00682	-4.9105	4.08E-38	3.10E-36
LINC00974	-4.9030	7.40E-61	3.15E-58
LINC01645	-4.6959	1.34E-37	9.48E-36
CDKN2B-AS1	-4.6555	2.12E-46	3.01E-44
HAND2-AS1	-4.5121	6.30E-27	1.84E-25
LINC01289	-4.3832	3.26E-11	2.42E-10
ADAMTS9-AS1	-4.3142	4.24E-26	1.13E-24
LINC00507	-4.2253	1.79E-34	9.27E-33
LINC00955	-4.1101	9.59E-15	9.54E-14
ERVMER61-1	6.4236	1.12E-08	5.99E-08
IGFL2-AS1	6.4603	6.05E-24	1.42E-22
AFAP1-AS1	6.5073	5.12E-32	2.06E-30
CASC21	6.5080	7.66E-49	1.36E-46
LINC01705	6.7817	1.40E-39	1.15E-37
LINC02163	6.8651	5.13E-50	1.36E-47
HULC	6.8808	6.05E-17	7.40E-16
CCAT1	6.9624	6.00E-42	5.81E-40
LINC01234	7.8465	6.56E-49	1.27E-46
FEZF1-AS1	9.1641	3.50E-46	4.65E-44

**Table 2:** Correlation between expression of CCAT1 and clinical pathology in 50 cases of colorectal cancer tissues.

**Pathological feature**		**LncRNA CCAT1**		***P*-value**
	**Sample amount**	**Low expression**	**High expression**	
Sex				
Male	25	13	12	
Female	25	11	14	0.5713
Age (y)				
<65	22	12	10	
≥65	28	13	15	0.5688
Tumor size				
≤3 cm	20	8	12	
>3 cm	30	17	13	0.2482
Location				
Left	31	16	15	
Right	19	9	11	0.6446
AJCC stage				
I-II	29	19	10	
III-IV	21	7	14	0.0246*
Lymph node metastasis				
N_0_	13	8	5	
N_1_	19	5	14	
N_2_	18	3	15	0.0242*
Distant metastasis				
M_0_	33	21	12	
M_1_	17	5	12	0.0218*

AJCC: American Joint Committee On Cancer. Chi-square test (*P<0.05).

### Targeting relationship between CCAT1 and miR-181a-5p in CRC cells

**Figure 2:**
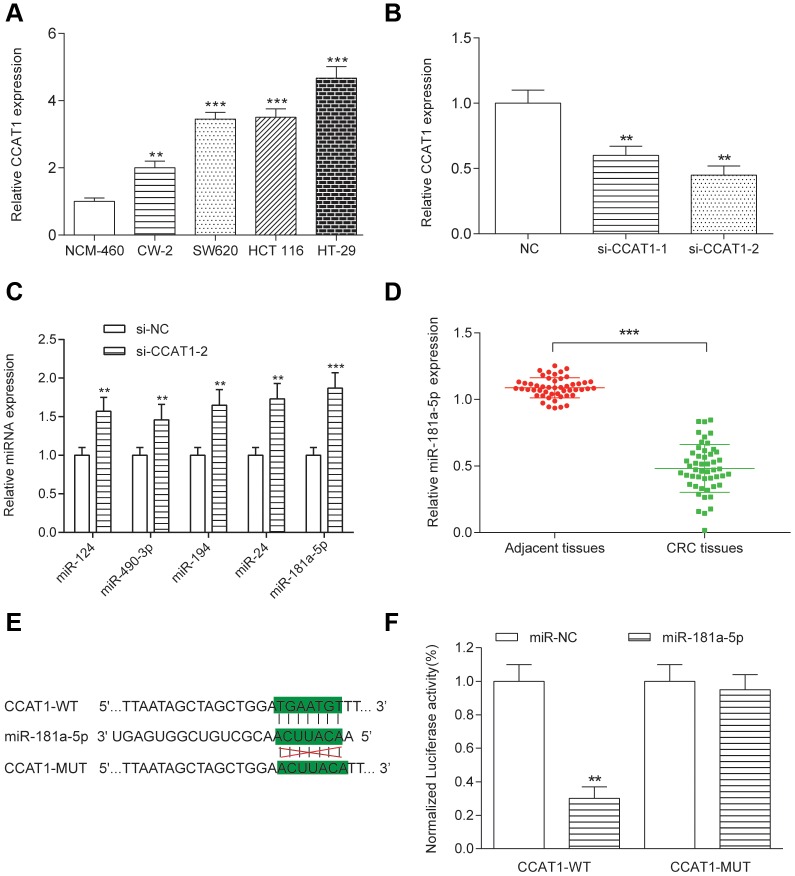
**Targeting relationship between CCAT1 and miR-181a in HT-29 cells.** (**A**) The CCAT1 expression levels in four CRC cell lines (CW-2, SW-620, HCT 116 and HT-29) and the human normal colorectal cell NCM-460 were detected by qRT-PCR. The expression of CCAT1 was normalized to that in NCM-460. (**B**) HT-29 was transfected with two different siRNAs against CCAT1. (**C**) miR-124, miR-490-3p, miR-194, miR-24 and miR-181a-5p expression in HT-29 cells. (**D**) MiR-181a-5p was downregulated in 50 paired CRC tissues and adjacent tissues. (**E**) Putative miR-181a-5p binding sequence of CCAT1 is shown. (**F**) The relative luciferase activity was detected in HT-29 cells co-transfected with CCAT1-WT or CCAT1-MUT and miR-181a-5p or miR-NC. All assays were performed three times. ^**^P<0.01, ^***^P <0.001, compared with NC group.

**Figure 3:**
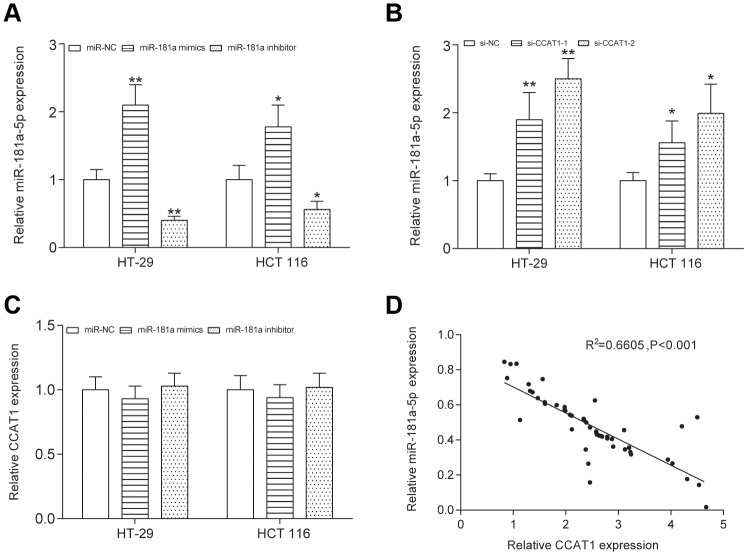
**CCAT1 and miR-181a-5p was negative correlative.** (**A**) The expression level of miR-181a-5p was upregulated by transfecting the miR-181a-5p mimics and was downregulated by transfecting the miR-181a-5p inhibitor into both HT-29 and HCT 116 cells. (**B**) After transfection of si-CCAT1-2 and si-CCAT1-1 in HT-29 and HCT 116 cells, the expression of miR-181a-5p was significantly upregulated. (**C**) Transfection of miR-181a-5p mimics or inhibitor could not reversely affect the expression of CCAT1. (**D**) The correlation between CCAT1 and miR-181a-5p expression level was measured in 50 CRC tissues. All assays were performed three times. ^**^P<0.01, compared with NC group.

To explore the biological functions of CCAT1 in CRC, we compared CCAT1 expression in four CRC cell lines (CW-2, SW620, HCT 116 and HT-29 cells) with a human normal colorectal cell line NCM-460. We found that expression level of CCAT1 was significantly upregulated in the tumor cell lines, particularly HT-29 cells (*P*<0.01, Figure 2A). HT-29 and HCT 116 cells were used in the later experiments. Furthermore, two different siRNAs oligos against CCAT1 were transfected into HT-29 and HCT 116 cells. Both siRNAs could efficiently knock down the endogenous CCAT1. Although the expression level of CCAT1 after si-CCAT1-2 transfection was much lower than that transfected by si-CCAT1-1, we used both si-CCAT1-1 and si-CCAT1-2 in the later experiments for efficiently silencing CCAT1 in order to obtain more reliable results (*P*<0.01, Figure 2B). We referred to miRcode (http://www.mircode.org/index.php) and then found that miR-124, miR-490-3p, miR-194, miR-24 and miR-181a-5p were potential targets of CCAT1. Expression analysis based on transcriptional profile showed that miR-181a-5p is the only candidate expresses inversely correlated with CCAT1 (Table 3). High level of miR-181a-5p was observed in cancer cell lines with knockdown of CCAT1 (Figure 2C). Low level of miR-181a-5p was found in CRC tissues (*P*<0.01, Figure 2D). The luciferase reporter assay in CRC cells further confirmed that wild-type CCAT1 contained a binding site for miR-181a-5p, but the mutant CCAT1 didn’t show the combining site for miR-181a-5p (*P*<0.01, Figure 2E, 2F, Supplementary Figure 5). MiR-181a-5p was significantly upregulated and downregulated after transfected miR-181a-5p mimics and miR-181a-5p inhibitor respectively in both HT-29 and HCT 116 cells (*P*<0.01, Figure 3A). After transfection of si-CCAT1-2 in HT-29 and HCT 116 cells, the expression of miR-181a-5p was significantly upregulated (*P*<0.01, Figure 3E, Supplementary Figure 6). However, transfection of miR-181a-5p mimics or inhibitor did not affect the expression of CCAT1 (Figure 3C). QRT-PCR results indicated a significant negative correlation between CCAT1 and miR-181a-5p (R^2^ = 0.6605, *P*<0.001, Figure 3D).

**Table 3:** Difference of the miRNA related to CCAT1.

**Gene symbol**	**log2FC**	**P.value**	**FDR**
hsa-mir-181a-5p	-3.089640896	9.99E-22	1.02E-20
hsa-mir-490	-2.033847066	0.0935452	0.136250623
hsa-mir-194-2	0.544711818	0.3059221	0.367472193
hsa-mir-24-1	1.896941381	7.82E-11	3.96E-10
has-mir-129-1	null	null	null

### Effects of CCAT1 and miR-181a-5p on the viability and proliferation of CRC cells

**Figure 4:**
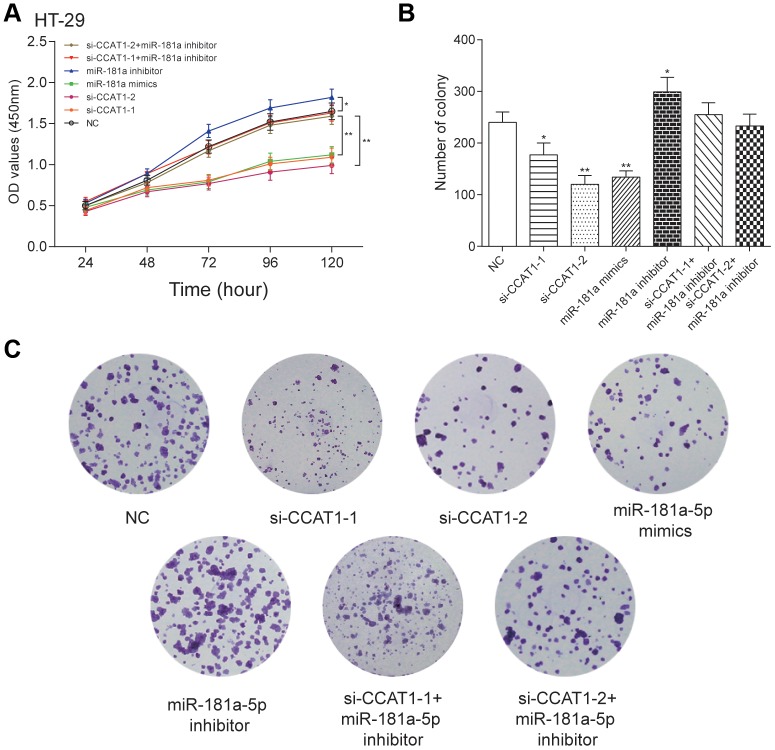
**LncRNA CCAT1 promoted proliferation of HT-29 cells by targeting miR-181a-5p.** (**A**) MTT assay demonstrated that depletion of CCAT1 inhibited cell proliferation. (**B**, **C**) The effect of si-CCAT1-1, si-CCAT1-2, miR-181a-5p mimics or miR-181a-5p inhibitor on CRC cell colony formation. ^*^P<0.05, ^**^P<0.01, compared with NC group

**Figure 5:**
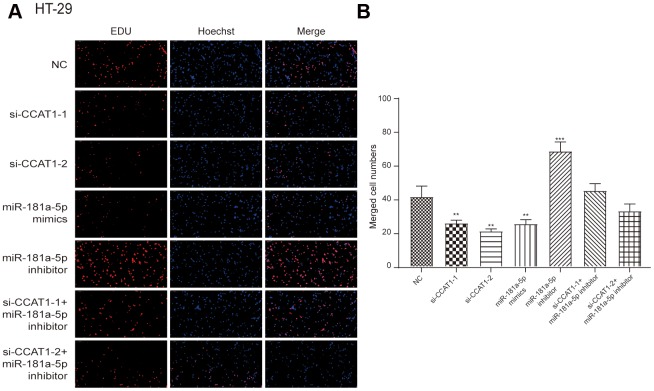
**EdU stain assay.** (**A**) The results showed that si-CCAT1-1, si-CCAT1-2 and miR-181a-5p mimics significantly inhibited the proliferation of HT-29 cells, whereas miR-181a-5p inhibitor significantly promoted cell proliferation. (**B**) The bar chart indicated that the merged cell number was significantly decreased after knock down of CCAT1 in HT-29 cell lines. All assays were performed three times.

By modulating the expression of CCAT1 and miR-181a-5p, we found that si-CCAT1-2, si-CCAT1-1 and miR-181a-5p mimics led to significantly lowered cell viability, whereas a stronger cell viability was seen in miR-181a-5p inhibitor group in both HT-29 and HCT 116 cell lines (*P*<0.05, Figure 4A and Supplementary Figure 1A). Similar results were seen in colony formation assay in two CRC cell lines (Figure 4B, 4C and Supplementary Figure 1B, 1C). EdU assay showed that si-CCAT1-2 and miR-181a-5p mimics significantly inhibited the proliferation of CRC cells, whereas miR-181a-5p inhibitor transfection group significantly increased the proliferation in CRC cells (Figure 5 and Supplementary Figure 2), suggesting that the cell viability and proliferation was inhibited by downregulation of CCAT1 or upregulation of miR-181a-5p.

### Effects of CCAT1 and miR-181a-5p on CRC cell aggression

**Figure 6:**
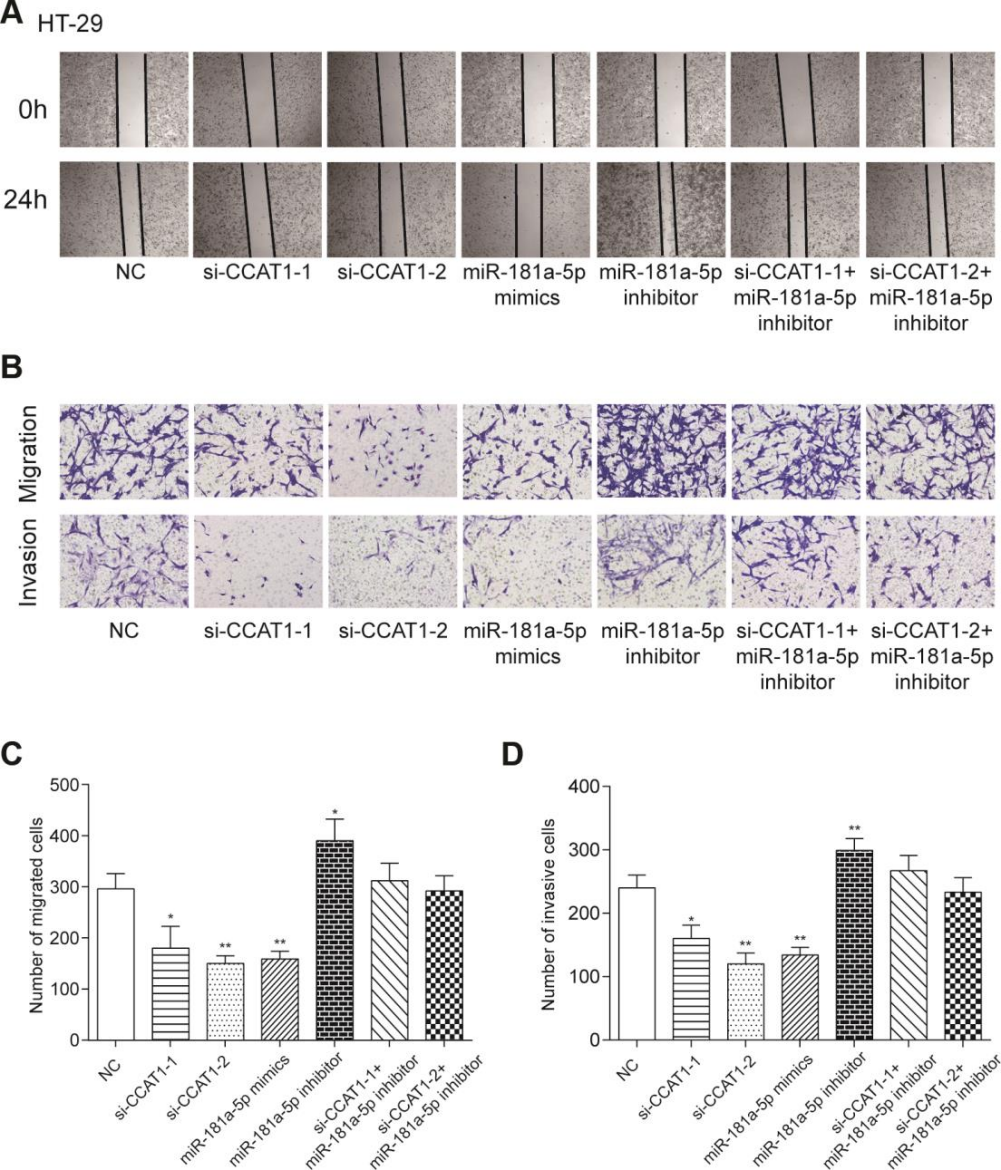
**LncRNA CCAT1 increased migration and invasion capabilities of CRC cells by targeting miR-181a-5p.** (**A**) Scratch-wound healing assay was used to assess the migration potency of HT-29 cells after being transfected. (**B**–**D**) Relative migration and invasion cell numbers of HT-29 cells transfected with si-CCAT1-1, si-CCAT1-2 or miR-181a-5p mimics detected by transwell assay significantly decreased. ^*^P<0.05, ^**^P<0.01, compared with NC group.

By modulating the expression of CCAT1 and miR-181a-5p, we found that si-CCAT1-1, si-CCAT1-2 and miR-181a-5p mimics led to significantly slower wound closure (Figure 6A and Supplementary Figure 3A). Transwell assays demonstrated that downregulation of CCAT1 and overexpression of miR-181a-5p impaired migration and invasion ability of CRC cells, while miR-181a-5p downregulation potentiated that ability in both of the two selected CRC cell lines (*P*<0.01, Figure 6B–6D, Supplementary Figure 3B–3D). These results implied that CCAT1 downregulation or miR-181a-5p upregulation was effective in suppressing the ability of cell migration and invasion.

### Effects of CCAT1 and miR-181a-5p on apoptosis of CRC cells

**Figure 7:**
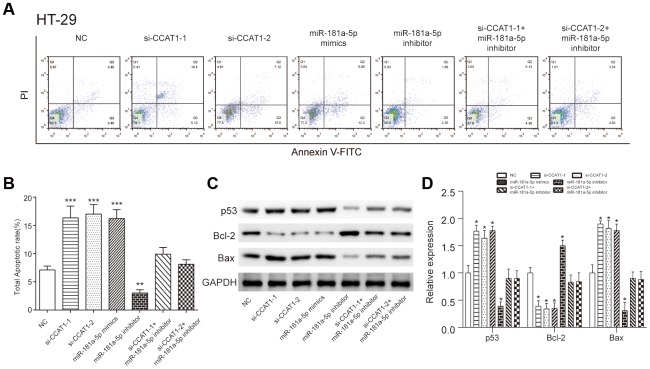
**LncRNA CCAT1 influenced colorectal cancer cell apoptosis through targeting miR-181a-5p.** (**A**, **B**) The apoptosis rate of HT-29 cells transfected with si-CCAT1-1, si-CCAT1-2 or miR-181a-5p mimics dramatically increased, whereas that of cells transfected with miR-181a-5p inhibitor remarkably decrease detected by flow cytometry. (**C**, **D**) Western blot analysis of p53 protein level and apoptosis-related protein Bax and Bcl-2 expression levels in HT-29 cells. The bar chart illustrated the level of p53 and Bax were significantly higher than NC group after CCAT1 knockdown and miR-181a-5p overexpression. All assays were performed three times. ^*^P<0.05, ^**^P<0.01^, ***^P<0.001, compared with NC group.

The apoptosis of HT-29 and HCT 116 cells was improved by downregulation of CCAT1. Similarly, cancer cells transfected with miR-181a-5p mimics also showed higher apoptosis rate (*P*<0.001, Figure 7A, 7B, Supplementary Figure 4A, 4B). Consistent with flow cytometry results, western blot analysis implied CCAT1 knockdown increased p53 and apoptosis-related proteins Bax expression levels, while Bcl-2 proteins expression level was decreased (Figure 7C, 7D, Supplementary Figure 4C, 4D). These results suggested that CCAT1 could regulate CRC cell apoptosis in part by modulating p53 expression.

### Knockdown of CCAT1 inhibited tumor growth in nude mice

**Figure 8:**
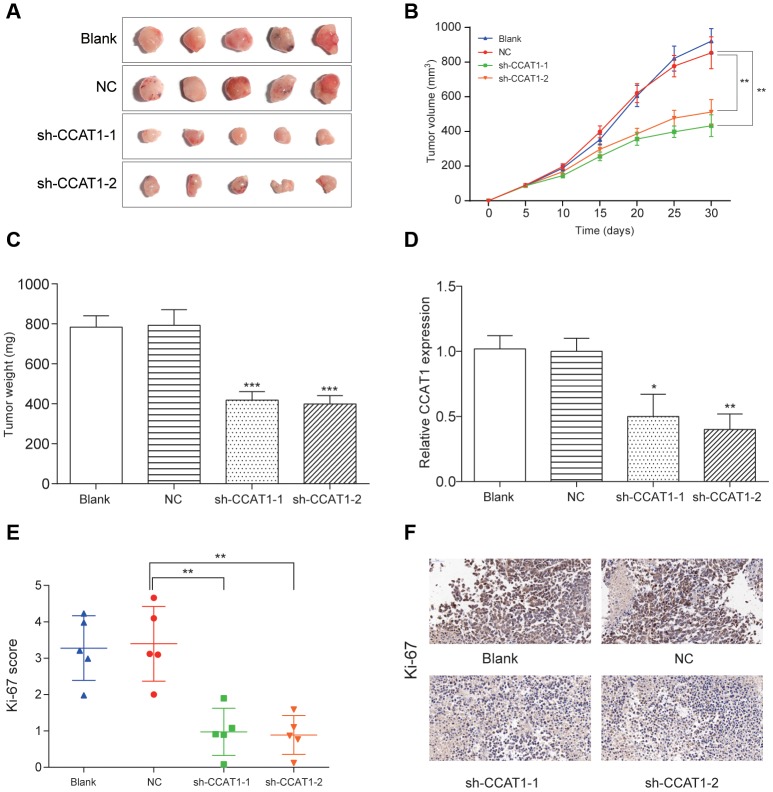
**LncRNA CCAT1 knockdown impaired colorectal cancer cell tumor growth *in vivo*.** (**A**–**C**) The *in vivo* tumor formation assay suggested that CCAT1 knockdown dramatically reduced tumor size and weight in 5 samples of each group. (**D**) The expression of CCAT1 was downregulated in resected tumor tissues formed from CCAT1 knockdown. (**E**, **F**) Immunohistochemistry showed CCAT1 knockdown decreased the proliferation index Ki67 (×50). ^*^P<0.05, ^**^P<0.01^, ***^P<0.001, compared with NC group.

Within 30 days after the xenograft model establishment, the average tumor volume and tumor weight in HT-29 cells with CCAT1 knockdown was reduced compared with mock cells (*P*<0.05, Figure 8A–8C). Moreover, the expression of CCAT1 detected by qRT-PCR assay was significantly downregulated in resected tumor tissues formed from CCAT1 knockdown, compared with those formed from NC group cells (*P*<0. 05, Figure 8D). Then using immunohistochemical staining of tumor tissues for Ki-67 detection, we found that the proliferation index was reduced in tumor tissues (*P*<0.01, Figure 8E, 8F). These results indicated that CCAT1 downregulation could inhibit tumor growth *in vivo*.

## DISCUSSION

Overexpression of CCAT1 and downregulation of miR-181a-5p were observed in CRC tissues and cell lines. Subsequent experiments verified that CCAT1 played a tumor-promoter role by down-regulating miR-181a-5p in CRC cells. Downregulation of CCAT1 or upregulation of miR-181a-5p suppressed the CRC cell proliferation, migration and invasion. Likewise, downregulation of CCAT1 or upregulation of miR-181a-5p accelerated cell apoptosis, which was verified in *in vivo* of nude mice that the tumor growth was suppressed by silencing of CCAT1.

A diversity of endogenous and extrinsic factors participated in CRC initiation and progression. Plenty of evidence has revealed ectopic expression of lncRNAs in CRC cells. Some studies have demonstrated that CCAT1 expression was significantly overexpressed in CRC patients compared with non-CRC patients [5, 19], which is consistent with the present study. The functions of these lncRNAs have been elementarily investigated. For instance, lncRNA-422 has been indicated to be a CRC suppressor [20]. By directly interacting with miR-125a-5p, lncRNA HOXA11-AS was found regulating CRC metastasis to liver [21]. Overexpression of lncRNA-ATB was significantly related with tumor growth, invasion, and lymph node metastasis [22]. LncRNA TUSC7 inhibits cell proliferation by targeting miR-211 in CRC [23]. Notably, forced overexpression of CCAT1 facilitated CRC cell proliferation and aggression [24], which was verified in our study. In addition, Kam et al*.* demonstrated that CCAT1 was exclusively expressed in CRC tissues as opposed to normal tissues [25]. However, marginal expression of CCAT1 in matched adjacent normal tissues were still detected in the present study.

Subsequently, we discovered the functional target of CCAT1, miR-181a-5p. Regulation function of miR-181a-5p was uncovered in a plenty of previous researches and reports. However, it is controversial about the role of miR-181a-5p in modulating CRC cell processes, including cell proliferation, migration, invasion and differentiation. miR-181a-5p was initially found under-expressed in CRC cells [26]. Our study detected low expression of miR-181a-5p in CRC cells and we verified that it could decrease cell proliferation, mobility and invasion, as well as accelerate cell apoptosis. that the importance role of p53 in cell apoptosis has been well-established [27]. In present study, downregulation of CCAT1 and upregulation of miR-181a-5p promoted cell apoptosis by regulating apoptosis-related proteins Bax and Bcl-2 via p53 signal pathway. Consistently, Lv et al*.* discovered that upregulation of miR-181a-5p suppressed cell viability and inhibited apoptosis of SW480 and LOVO cells by suppressing expression of ZEB1-AS1 [28]. However, as Zhang et al*.* pointed out, the forced expression of miR-181a-5p enhanced CRC cell proliferation [29]. Ji et al*.* demonstrated that miR-181a-5p enhanced tumor growth and liver metastasis in CRC by targeting tumor suppressor *WIF-1* [30]. These results seem contradictory to ours. However, cell context could contribute to the difference. Zhang et al*.* used LoVo and SW480, and Ji et al*.* used only RKO and LOVO cell lines to force express miR-181a-5p.

Recently, a new mechanism underlying the regulatory relation between lncRNA and miRNA has been proposed where they act as competing endogenous RNAs, also known as ceRNAs. ceRNAs are involved in a variety of biological process especially in the processes of tumorigenesis [31, 32]. In our study the CCAT1 and miR-181a-5p might serve as ceRNAs and our results indicated that they could affect the growth of CRC tumor by regulating the p53 signaling pathway. Together, our findings promise the potential value of a new therapeutic regime that harnesses ceRNAs to mitigate the progression of CRC.

Despite the full investigation of miR-181a-5p-associated mechanism in CRC, we hypothesize that the miR-181a-5p, targeted by CCAT1, might regulate the proliferation, migration, and invasion of CRC through the p53 signaling pathway, which warrants further exploration of the relationship between miR-181a-5p level and pathological characteristics.

## CONCLUSIONS

Our study demonstrated that abnormal expression of miR-181a-5p regulated by CCAT1 may be correlated with CRC development and progression. Knockdown of CCAT1 inhibited CRC cell proliferation and aggression by targeting miR-181a-5p. Simultaneously, suppressing CCAT1 expression or increasing miR-181a-5p expression substantially accelerated CRC cell apoptosis by modulating p53 protein. These results illuminate a prospective therapeutic regime for CRC. However, abundant tumor-associated proteins are potential target of CCAT1 and miR-181a-5p, therefore, more functionalities of CCAT1 remain to be explored.

## MATERIALS AND METHODS

### Clinic samples

Fifty paired colorectal cancer tissues and adjacent tissues were collected from patients who underwent surgery at the Tongji Hospital of Tongji University School of Medicine between January 2014 and December 2017. The selection criteria excludes those patients who underwent any preoperative radiotherapy or chemotherapy before blood collection; with inflammatory bowel disease, hereditary colorectal cancer, or other rare tumor types; or were unable to provide informed consent. All enrolled cases were histopathologically confirmed and obtained with informed consent. The study was ratified by the Ethics Committee of Tongji Hospital of Tongji University School of Medicine. No patient received preoperative local or systemic anticancer treatment. Tumor stage was classified based on the guideline of Cancer Staging Manual of TNM of the 8^th^ Edition of the American Joint Committee on Cancer.

### Microarray analysis

RNA sequencing (RNA-Seq) data from 521 individuals with COAD were obtained from TCGA Data Portal Bulk Download (https://tcga-data.nci.nih.gov/tcga), including data from 366 COAD tissue samples and 155 non-tumorous adjacent-normal colorectal tissue samples. We obtained the expression profiles of 2,128 lncRNAs using 38 pairs of CRC tissues and matched adjacent tissues. We then filtered the differentially expressed lncRNAs using two individual R packages: edgeR and DEseq, with FDR<0.05 and |log_2_FC (fold change)|>1 of expression level between comparison of tumor and adjacent normal colorectal tissue. Finally, we identified 20 differentially expressed lncRNAs including CCAT1 to plot the heatmap.

### Cell culture

Human normal colorectal cell line and CRC cell lines were purchased from BeNa Culture Collection (Beijing, China). Human CRC cell lines included CW-2, SW-620, HCT 116 and HT-29. SW-620 and HCT 116 cell lines were cultured in RPMI-1640 containing 10% fetal bovine serum (FBS, Beinuo life science, Shanghai, China). CW-2 and HT-29 were cultured in RPMI-1640 containing 10% FBS. The normal colorectal cell line NCM-460 was cultured in McCoy’s 5A medium containing 10% FBS (Shanghai Bioleaf Biotech Co. Ltd, Shanghai, China).

### Quantitative real time polymerase chain reaction (qRT-PCR)

qRT-PCR was performed as previously described [33]. Briefly, reverse transcription was performed with the reverse transcription kit (Solarbio, Shanghai, China). Real-time fluorescence PCR was performed using SYBR Green quantification kit (Solarbio). The levels of CCAT1 and miR-181a-5p were normalized to GAPDH mRNA using the 2^-ΔΔCt^ method. PCR primers were designed as Table 4.

**Table 4:** Primer sequence.

	**Primer sequence**
miR-181a forward	5'-ACACTCCAGCTGGGTAACATTCAACGCTC-3'
miR-181a reverse	5'-CTCAACTGGTGTCGTGGA-3'
U6 forward	5'-CTCGCTTCGGCAGCACATA-3'
U6 reverse	5'-AACGATTCACGAATTTGCGT-3'
CCAT1 forward	5'-TTTATGCTTGAGCCTTGA-3'
CCAT1 reverse	5'-CTTGCCTGAAATACTTGC-3'
GAPDH forward	5'-GAAGGTGAAGGTCGGAGTC-3'
GAPDH reverse	5'-GAAGATGGTGATGGGATTTC-3'

### Cell transfection

CRC cells for *in vitro* assays were transfected with NC, si-CCAT1 (si-CCAT1-1, si-CCAT1-2), miR-181a-5p mimics, miR-181a-5p inhibitor, si-CCAT1-2+miR-181a-5p inhibitor. MiRNA inhibitor is small, chemically modified single-stranded RNA molecules designed to specifically bind to and inhibit endogenous miRNA molecules. And miRNA inhibitor acts like molecular sponge, soak up miRNAs, titrate miRNA activity which competitively bind to their target miRNA through complementarity to seed sequences [34, 35]. All small molecules in this experiment were purchased from GenePharma (Shanghai, China). All transfections were performed using by Lipofectamine 3000 (purchased from Solarbio). 48 hours post transfection, cells were harvested for transfection efficiency test. CRC cells for *in vivo* assays were transfected with NC, sh-CCAT1 (sh-CCAT1-1, sh-CCAT1-2). The transfections were performed using by Lenti-Pac™ HIV expression packaging kit (GeneCopoeia, Inc., Guangdong, China). All detailed procedures were followed by the manufactures’ instructions. The sequences were shown in Table 5.

**Table 5:** Sequence of siRNA and shRNA.

	**Sequence**
si-CCAT1-1 sense	5'-AAAGGTGCCGAGACATGAA-3'
si-CCAT1-1 antisense	3'-TTTCCACGGCTCTGTACTT-5'
si-CCAT1-2 sense	5'-AGGCAGAAAGCCGTATCTT-3'
si-CCAT1-2 antisense	3'-TCCGTCTTTCGGCATAGAA-5'
sh-CCAT1-1 sense	5'-GGCGATAGACGACGGATTGAT-3'
sh-CCAT1-1 antisense	5'-ATCAATCCGTCGTCTATCGCC-3'
sh-CCAT1-2 sense	5'-GGCTGGAGAGCAGATAGGTAT-3'
sh-CCAT1-2 antisense	5'-ATACCTATCTGCTCTCCAGCC-3'
miR-181a inhibitor	5'-ACUCACCGACAGCGUUGAAUGUU-3'

### Western blot

The electrophoretic separation with 10% dodecyl sulfate-sodium salt-polyacrylamide gel electrophoresis was conducted with each lane loaded with 50 μg of total proteins. The separated proteins were then blotted to polyvinylidene fluoride membranes. Subsequently, the membranes were subjected to incubation with primary antibodies of p53 (5 μg/ml, #ab1101), Bax (1:1000, #ab32503), Bcl-2 (1:1000, #ab32124) and GAPDH (1:1000, #ab8245) overnight at 4°C, and goat anti-rabbit HRP horseradish peroxidase-labeled antibodies (1:2000, #ab6721) for 1 h. All antibodies were purchased from Abcam, Cambridge, UK.

### Immunohistochemistry

Fresh tissues were cut into 4 μm thick slices, which were subsequently subjected to immunostaining. The slices were incubated with Ki-67 antibody (#ab15580) overnight at 4 °C. Histostain-Plus 3^rd^ Gen IHC Detection Kit (Invitrogen Co, San Diego, CA) was applied for 30 min to visualize the positive signals.

### Luciferase reporter gene assay

The HT-29 and HCT 116 cells (2.0×10^4^) grown in a 96-well plate were co-transfected with 150ng of empty pmir-GLO-NC, pmir-GLO-CCAT1-Wt, or pmir-GLO-CCAT1-Mut (Sangon Biotech, China) and 2 ng of pRL-TK (Promega, Madison, WI, USA) with a miR-181a-5p mimic or miR-NC into cells using Lipofectamine 3000 (Invitrogen, USA). 48 h after transfection, the relative luciferase activity was calculated by normalizing firefly luciferase to renilla luciferase.

### Nude mice experiments

The animal experiments were approved by the committee of Use of Animal Care in Tongji Hospital of Tongji University School of Medicine. HT-29 and HCT 116 cells with successful transfection of sh-CCAT1-1, sh-CCAT1-2 (two independent shRNAs, the sequences were showed in the Table 2), Blank and NC (approximately 1×10^6^ cells/mouse) were subcutaneously injected into the armpit of 6-week-old BALB/c athymic nude mice (five of each group, male vs. female: 3:2). Tumor volume and weight were measured at 5, 10, 15, 20, 25 and 30 days. The mice were euthanized 30 days after injection, and photographs of excised tumors were obtained. The excised tumors were then used into further analyses.

### Cell viability assay

For MTT assay, 20 μl MTT (Beyotime, Shanghai, China) was added to cells of each well. The optical absorbance was measured at 570 nm. For colony formation assay, 1,000 cells were plated into each well of a 6-well plate and were maintained in media containing 10% FBS to allow colony formation, with the medium being replaced every four days. In approximately 2 weeks, the colonies were fixed with methanol and stained with 0.1% crystal violet for 15 min. The stained colonies were counted. For EdU assay, cells were incubated with 50 μM EdU labeling/detection kit (RiboBio Co.) for 12 h. After the fixation with 4% paraformaldehyde for 30 min and incubated in 5% glycine for 5 min, the cells were incubated with anti-EdU antibody for 30 min. Lastly, the cells were stained with 5 μg/ml Hoechst 33342 for 0.5 h.

### Aggressiveness assay

For wound healing assay, 24 h post transfection, artificial wounds were made by scraping with a sterilized 200 μL pipette tip, and the debris was washed away with PBS. Cells were incubated with serum-free medium for a further 24h and were imaged after 24h using an inverted microscope. For Transwell assay, 36 h post transfection, HT-29 and HCT 116 cells were grown in the upper transwell chambers coated with Matrigel (Haoranbio, Shanghai, China) while 100 μL 10% FBS was added to the lower chambers separately. The upper chambers were soaked in the lower chambers for 24 h. Then the upper chambers were taken out and fixed by paraformaldehyde. The cells on the lower side of the membrane of upper chambers were stained by crystal violet for 30 min. 5 fields of each lower side of the membrane were photographed and the number of cells were counted.

### Cell apoptosis assay

FACScan flow cytometer (BD Biosciences, USA) was used to determine cell apoptosis. The cells were stained by propidium iodide (PI) and Annexin V (BestBio, China).

### Statistical analysis

All experiments were performed at least in triplicate. All data were analyzed with GraphPad Prism 6.0. Continuous data were displayed as mean ± standard deviation, in which the differences within two different groups were analyzed by Student's t-test while one-way ANOVA were performed to analyze the difference among multiple groups. *P* value < 0.05 was regarded as statistically significant.

## Supplementary Material

Supplementary Figures
