# Supplementary material for: Long non-coding RNA CCAT1 promotes colorectal cancer progression by regulating miR-181a-5p expression
Source: Aging (Albany NY). 2020 May 7;12(9):8301–20. doi: 10.18632/aging.103139 (PMC7244037; doi:10.18632/aging.103139)
Supplement: Supplementary Figures [file aging-12-103139-s001..pdf]

SUPPLEMENTARY FIGURES

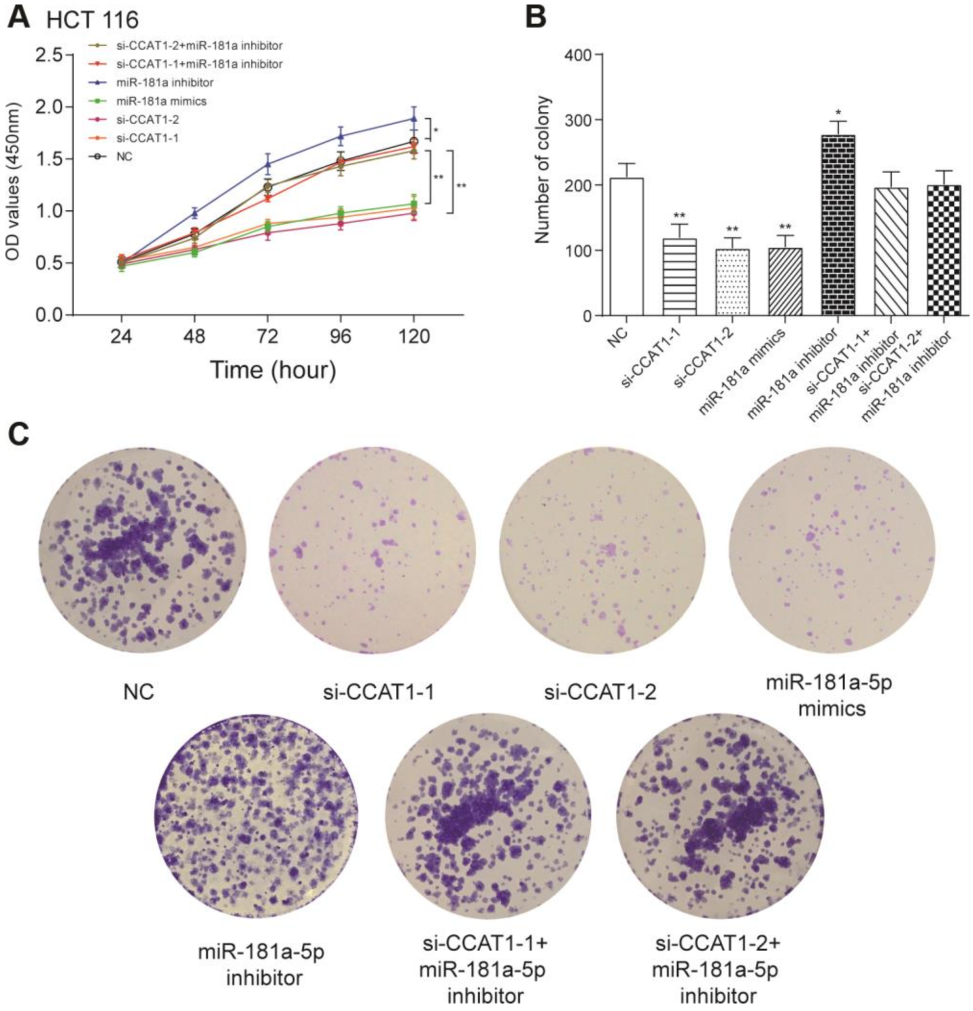

Supplementary Figure 1. LncRNA CCAT1 promoted proliferation of HCT 116 cells by targeting miR-181a-5p. (**A**) MTT assay demonstrated that depletion of CCAT1 inhibited cell proliferation. (**B**, **C**) The effect of si-CCAT1-1, si-CCAT1-2, miR-181a-5p mimics or miR-181a-5p inhibitor on CRC cell colony formation. $^{*}P<0.05$, $^{**}P<0.01$, compared with NC group

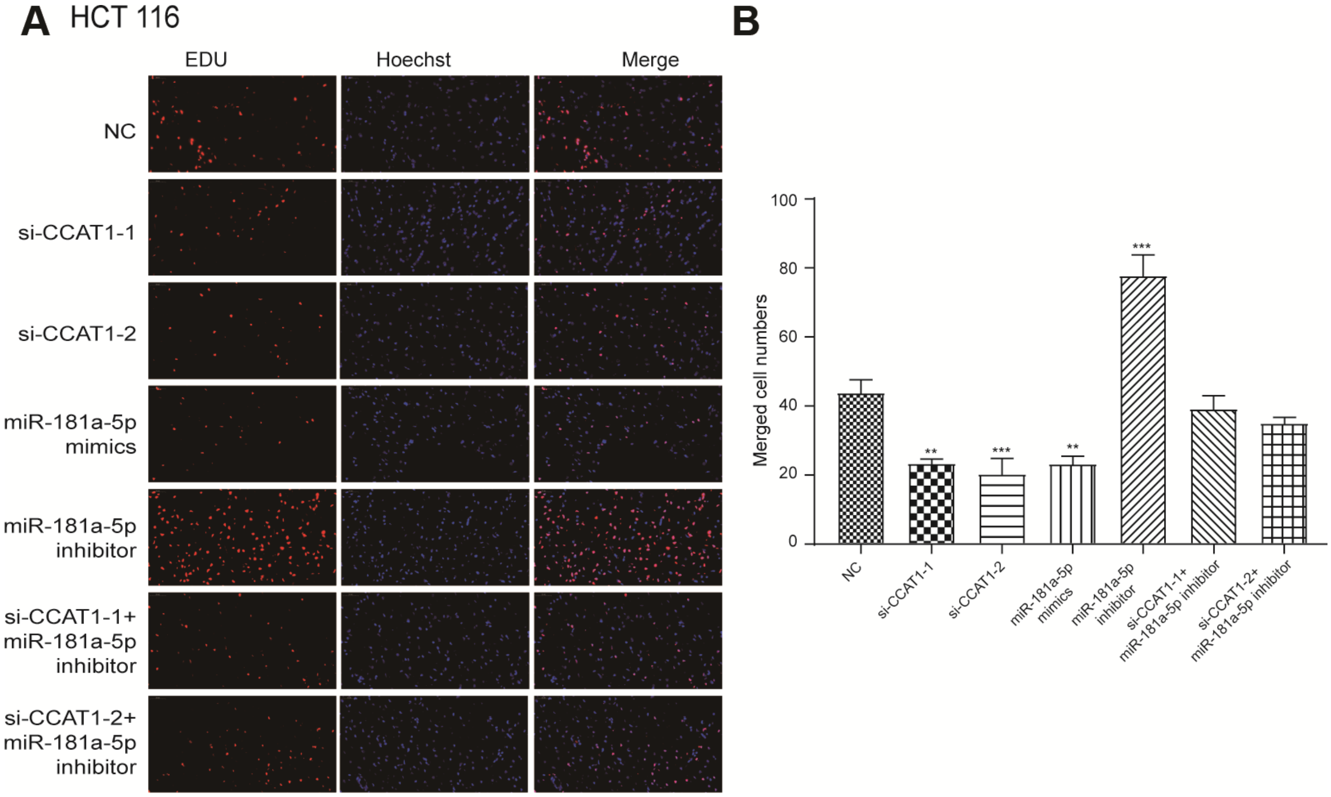

Supplementary Figure 2. EdU stain assay of HCT 116 cells. (**A**) The results showed that si-CCAT1-1, si-CCAT1-2 and miR-181a-5p mimics significantly inhibited the proliferation of HCT 116 cells, whereas miR-181a-5p inhibitor significantly promoted cell proliferation. (**B**) The bar chart indicated that the merged cell number was significantly decreased after knock down of CCAT1 in HCT 116 cell lines. All assays were performed three times.

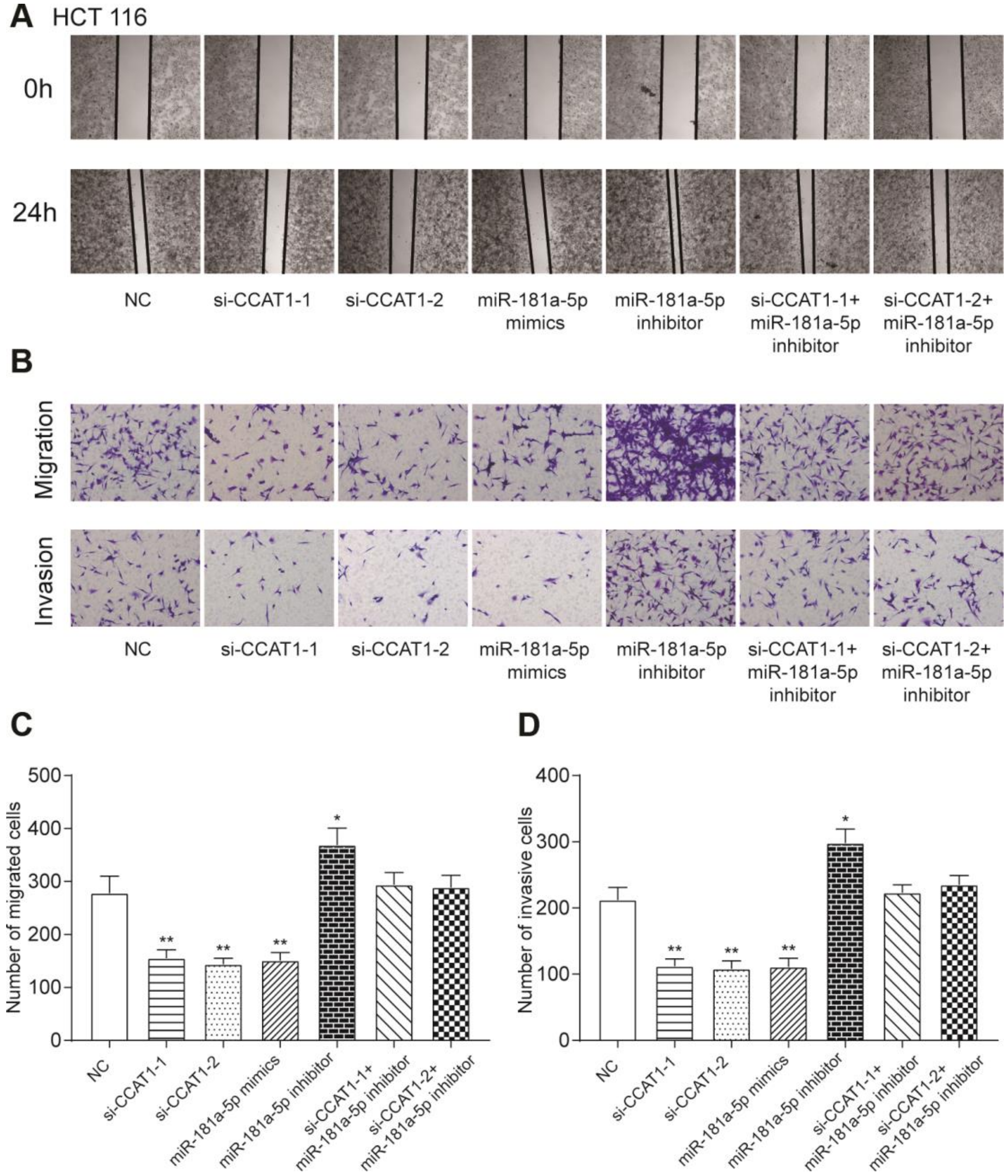

Supplementary Figure 3. LncRNA CCAT1 increased migration and invasion capabilities of CRC cells by targeting miR-181a-5p in HCT 116 cell lines. (**A**) Scratch-wound healing assay was used to assess the migration potency of HCT 116 cells after being transfected. (**B**–**D**) Relative migration and invasion cell numbers of HCT 116 cells transfected with si-CCAT1-1, si-CCAT1-2 or miR-181a-5p mimics detected by transwell assay significantly decreased. $^{*}P$<0.05, $^{**}P$<0.01, compared with NC group.

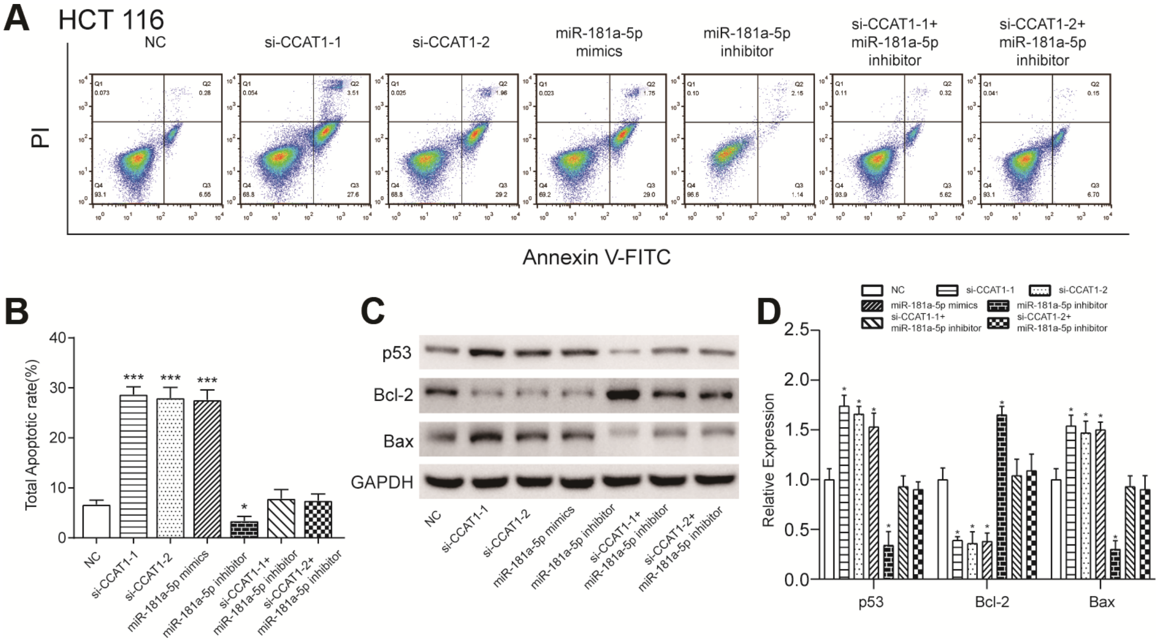

Supplementary Figure 4. LncRNA CCAT1 influenced colorectal cancer cell apoptosis through targeting miR-181a-5p. (**A**, **B**) The apoptosis rate of HCT 116 cells transfected with si-CCAT1-1, si-CCAT1-2 or miR-181a-5p mimics dramatically increased, whereas that of cells transfected with miR-181a-5p inhibitor remarkably decrease detected by flow cytometry. (**C**, **D**) Western blot analysis of p53 protein level and apoptosis-related protein Bax and Bcl-2 expression levels in HCT 116 cells. The bar chart illustrated the level of p53 and Bax were significantly higher than NC group after CCAT1 knockdown and miR-181a-5p overexpression. All assays were performed three times. $^{*}P$<0.05, $^{**}P$<0.01, $^{***}P$<0.001, compared with NC group.

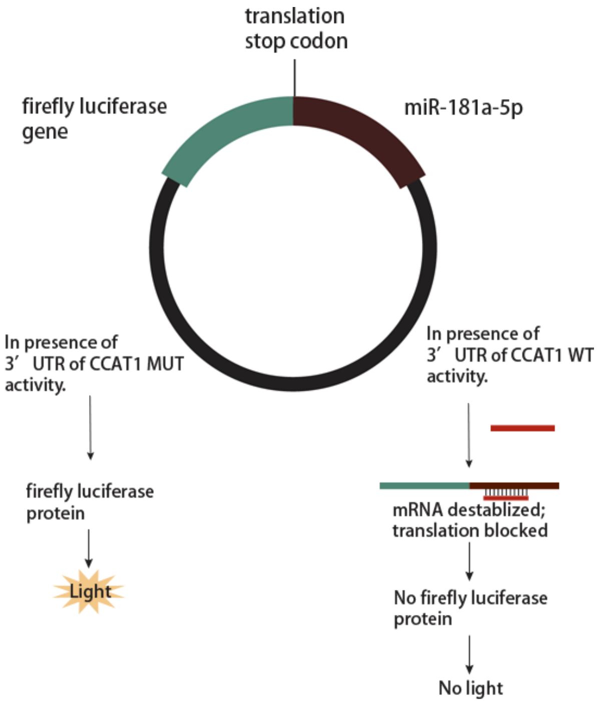

Supplementary Figure 5. Schema of mechanism of pmiR-GLO vector used for luciferase reporter assay to elucidate the targetability of miR-181a-5p by CCAT1.

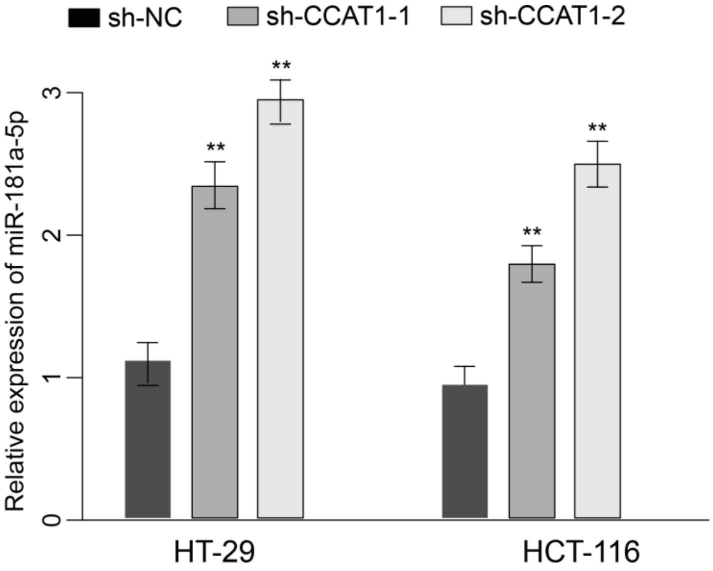

Supplementary Figure 6. Relative miR-181a-5p in HT-29 and HCT 116 cells transfected with sh-CCAT1-1/-2.
